# Automatic Railway Traffic Object Detection System Using Feature Fusion Refine Neural Network under Shunting Mode

**DOI:** 10.3390/s18061916

**Published:** 2018-06-12

**Authors:** Tao Ye, Baocheng Wang, Ping Song, Juan Li

**Affiliations:** 1 Beijing Institute of Remote Sensing and Equipment, 52 Yongding Road, Haidian District, Beijing 100039, China; 2 College of Computer and Science Technology, North China University of Technology, 5 Jin Yuan Zhuang Road, Shijingshan District, Beijing 100144, China; wbaocheng@ncut.edu.cn; 3 School of Instrumentation Science and Opto-Electronics Engineering, Key Laboratory of Precision Opto-Mechatronics Technology, Ministry of Education, Beihang University, Beijing 100191, China; zenghui_s@163.com (P.S.); sy1617314@buaa.edu.cn (J.L.)

**Keywords:** shunting mode, feature fusion refine neural network, depthwise-pointwise convolution, effectiveness and real time

## Abstract

Many accidents happen under shunting mode when the speed of a train is below 45 km/h. In this mode, train attendants observe the railway condition ahead using the traditional manual method and tell the observation results to the driver in order to avoid danger. To address this problem, an automatic object detection system based on convolutional neural network (CNN) is proposed to detect objects ahead in shunting mode, which is called Feature Fusion Refine neural network (FR-Net). It consists of three connected modules, i.e., the depthwise-pointwise convolution, the coarse detection module, and the object detection module. Depth-wise-pointwise convolutions are used to improve the detection in real time. The coarse detection module coarsely refine the locations and sizes of prior anchors to provide better initialization for the subsequent module and also reduces search space for the classification, whereas the object detection module aims to regress accurate object locations and predict the class labels for the prior anchors. The experimental results on the railway traffic dataset show that FR-Net achieves 0.8953 mAP with 72.3 FPS performance on a machine with a GeForce GTX1080Ti with the input size of 320 × 320 pixels. The results imply that FR-Net takes a good tradeoff both on effectiveness and real time performance. The proposed method can meet the needs of practical application in shunting mode.

## 1. Introduction

With the growth of railway transportation, increasing attention has been paid to railway safety. Moreover, with the development of artificial-intelligence technology, intelligent transportation system (ITSs) have increased in popularity to provide traffic safety [1,2,3,4,5]. ITSs are generally divided into intelligent infrastructure systems and intelligent vehicle systems. Although railways bring us convenience, they also experience many traffic accidents each year. In research on railway transportation, many scholars focus on infrastructure systems [5,6,7,8]. Some researchers believe that accidents within railway crossing boundaries are often caused when an approaching train collides with intruding pedestrians or vehicles that are on the tracks at the crossing [9,10,11,12]. However, many accidents happen under shunting mode when the speed of a train is below 45 km/h. In this mode, train attendants observe the railway condition ahead by using the traditional manual method and tell the observation results to the driver to avoid danger. Human error and fatigue reduce the safety of shunting operation, which increases the likelihood of shunting accidents and endangers the safety of persons and property [13,14,15,16]. With the rapid development of artificial intelligence, it is appropriate for machine vision detection methods to replace the traditional manual methods in shunting operations. This study focuses on detecting obstacles in shunting operations.

**Figure 1:**
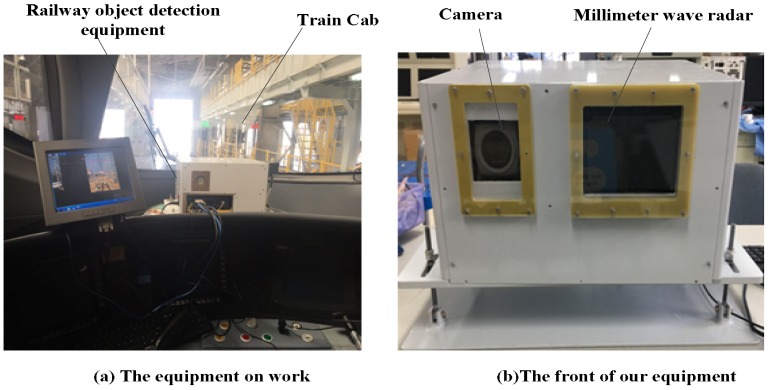
Principle prototype of our equipment.

With the aid of a graphics processing unit (GPU) hardware platform and convolutional neural networks (CNNs) [17,18], we designed a novel object detection system for a train to automatically detect objects that are ahead in shunting mode. The principle prototype equipment is shown in Figure 1a,b. We installed our principle prototype equipment on a train cab to capture railway traffic images. The equipment was developed by our project team, and it included a camera and a millimeter-wave radar—see Figure 1b. The camera collected images for our object detection algorithm, and the millimeter-wave radar measured the distance between the equipment and obstacles. The motivation of our detection system is to help train drivers to drive safely. The alarm part is used to send voice to remind the train attendants. When the train attendants are tired and are unable to concentrate, our detection system can inform the danger, such as a train ahead of the railway by voice prompt. In this work, six kinds of objects are detected, including railway straight, railway left, railway right, pedestrian, bullet train, and safety helmet. The motivation of detecting railway straight, left and right is to determine whether the train is running at the bend railway. If our system detects the bend railway, it reminds the driver of the train to drive safely by voice automatically. Meanwhile, the motivation of detecting of pedestrians (mostly for railway workers) and the front train is to let the train attendant find the possible danger ahead in time. When the proposed system detects the pedestrians or train on the railway ahead, the train attendants are informed by the voice prompt and take the corresponding measures to avoid the possible danger. As one of the objects that workers often leave on the railway, the detection of the safety helmet is to reduce unnecessary losses. In this study, we focused on railway traffic obstacle detection. The core of the equipment was the design of the feature fusion refine neural network (FR-Net) to detect obstacles on railways. In this work, we discuss the FR-Net in detail. We introduce depthwise-pointwise convolution [19] to improve the real-time performance of FR-Net. We adopt the thinking of the classical two-stage method (i.e., Faster rcnn [20]) for effective object detection. Unlike Faster rcnn, we employ a feature map and feature fusion to construct robust features for further object detection. In contrast to the conventional single-shot detector (SSD) [21], which directly uses regularly tiled default boxes for detection, FR-Net uses a two-step strategy: the coarse detection module generates the prior anchor boxes, and the object detection module takes the prior anchor boxes as input for further detection, leading to more-accurate detection results.

Our main contributions are as follows:

1. To account for effectiveness and efficiency, three novel parts were introduced in FR-Net, including depthwise convolution, the coarse detection module, and the object detection module. The coarse object detection module provided prior anchors for the object detection module. Taking the prior anchor boxes as the input, the object detection module obtained sufficient feature information using two submodules (i.e., the feature map fusion module and feature fusion module) for object detection. Depthwise convolution was used for efficiency, whereas the other two modules were responsible for effectiveness.
2. With input sizes of 320×320 test images, FR-Net achieves 0.89 mAP with performance of 72.3 frames per second (FPS). The experimental results show that FR-Net balances effectiveness and real-time performance well. The robustness experimental results show that the proposed model can conduct all-weather detection effectively in railway traffic situations. Moreover, the proposed method yields superiority over the SSD for small-object detection.

The rest of this work is organized, as follows. We discuss previous research on railway obstacle detection in Section 2. In Section 3, we introduce the proposed method. In Section 4, the experimental results and the performance analysis are discussed. We draw our conclusions in Section 5.

## 2. Related Work

### 2.1. Railway Obstacle Detection Systems

There has been significant previous research about railway traffic alerts and collision avoidance systems. The German Aeronautics and Astronautics Center has successfully demonstrated and verified the current research results of railway collision avoidance system (RCAS) theory on actual railway trains at the WegBerg railway laboratory base. RCASs can predict the danger ahead for the train driver and can used as equipment for the safe operation of the train. India Railway Co., Ltd. (Konkan, Indian) cooperated to produce the AntiCollision Device Network (ACDN) [22]. The ACDN system uses GPS to locate trains, and the trains identify and communicate with each other through radio. Liu [23] developed a millimeter-wave collision avoidance radar system for transportation safety. These methods used signal transfer to detect objects ahead, which can easily be disturbed by external signals and cannot distinguish the obstacles ahead. With the development of computer vision, some devices using image-processing technology have been introduced for railway object detection. Silar [24] studied detection that is based on optical-flow estimation and classification of railway-crossing objects by a K-means clustering algorithm. Yong [9] used machine vision to detect obstacles at railway crossings. Ryuta [25] proposed a method using a monocular camera and image processing for obstacle detection. For the methods that are based on image processing or conventional machine-learning techniques, it is difficult to design a unified method to detect and recognize various objects simultaneously. In this work, we developed a device that is mounted on a train using the proposed CNN architecture FR-Net to detect obstacles on railways.

### 2.2. Object Detection with CNNs

Prior to the convolutional neural networks (CNNs), different machine-learning algorithms were developed to improve object detection performance [26,27,28]. Some of the scholars [29,30] proposed algorithms that conduct background modeling and then detect the moving objects in the foreground. These algorithms are more suitable for security monitoring, while the background changes very little. However, the CNN methods detect the background and the moving objects for each sequential frame of a video, which are suitable for object detection in dynamic background. However, CNN approaches have recently been successful in the area of object detection [31]. The CNN-based detectors can be roughly divided into the two-stage approach and the one-stage approach. The two-stage approach detects objects using two steps. The first step [32,33] generates a set of candidate object proposals, and the second step determines the accurate object location and class labels using CNNs. The classic two-stage approaches, such as rcnn, Fast rcnn, Faster rcnn [34], and SPPnet [35], achieve dramatic improvements in accuracy. However, one problem with these kinds of method is that, to process many proposals, the computation in the second stage is usually heavy. To improve the efficiency, the one-stage approach is attracting increasing interest. The OverFeat [36] method applies a ConvNet as a feature extractor in the sliding window on an image pyramid, which is trained end-to-end, from pixels to classification. SSD [21] and YOLO [37] use a single feed-forward convolution network to directly predict object classes and locations, which are trained end-to-end. YOLO is extremely fast, with relatively low accuracy. SSD focuses on detecting objects of different scales by multiple layers within a ConvNet. A deconvolutional single-shot detector (DSSD) [38] introduces additional context into SSD via feature map fusion to improve accuracy. To improve accuracy [39,40], some of the one-stage approaches address the class imbalance problem by introducing a modified loss function and novel classification strategies. To improve the real-time performance, depthwise separable convolutions (e.g., inception models [41], factorized networks [42], and MobileNet [19]) are introduced to achieve excellent performance in the resource and accuracy tradeoff.

In this study, FR-Net, which is a two-stage detector, is introduced. It inherits the merits of Faster rcnn, SSD, and DSSD, which can detect objects effectively; meanwhile, it can obtain good real-time performance by introducing depthwise–pointwise convolution.

## 3. Proposes Method

**Figure 2:**
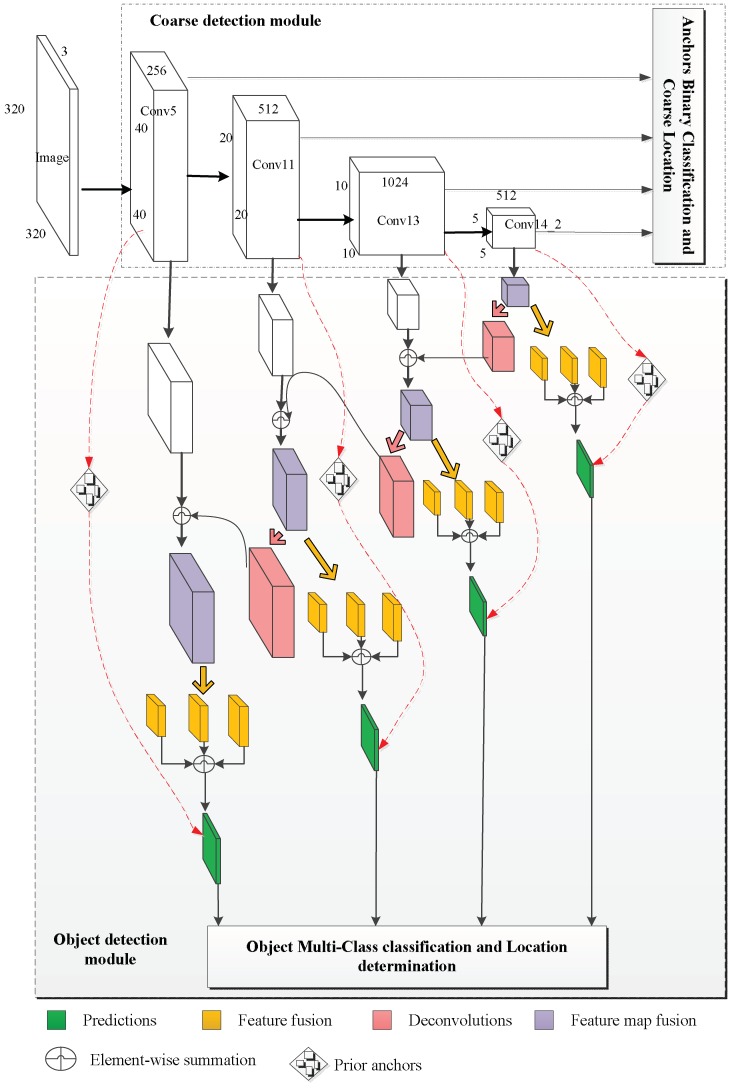
Feature Fusion Refine neural (FR-Net) architecture overview includes depthwise–pointwise convolutions, a coarse object detection module, and an object detection module. Predictions are carried out by four green layers with different scales. Different colors represent different function layers.

The FR-Net is introduced for solving the problem of real-word railway traffic object detection. For practical application, we emphasize the real-time performance and effectiveness of FR-Net. The FR-Net architecture is shown in Figure 2. Like SSD, FR-Net is based on a feed-forward convolutional network that produces a fixed number of anchor boxes and computes the scores of the objects belonging to different classes. Then, non-maximum suppression is used to produce the final result. FR-Net is formed by three connected modules, i.e., depthwise-pointwise convolution, the coarse detection module, and the object detection module. Depthwise-pointwise convolutions are used to improve the detection in real time, whereas the other two modules are utilized for the effectiveness of the network. The coarse detection module is constructed by removing the classification layers and adding the auxiliary structure of a base network (VGG-16 pretrained on ImageNet in this work) to meet our needs. Furthermore, we replace all of the standard convolutions with depthwise-pointwise convolutions, except for the first layer, which can make a deep neural network lightweight. The coarse detection module coarsely refines the locations and sizes of prior anchors to provide better initialization for the subsequent module, and it also reduces search space for classification, whereas the object detection module aims to regress accurate object locations and to predict the class labels for the prior anchors. The object detection module is composed of the outputs of feature map fusion and feature fusion modules followed by prediction layers, which generate the scores for object classes and location offset coordinates relative to the refined anchor box. In this work, feature layers of conv5 (size of 40 × 40 with 256 channels), conv11 (size of 20 × 20 with 512 channels), conv13 (size of 10 × 10 with 1024 channels), and conv14_2 (size of 5 × 5 with 512 channels) are considered as a basic element to conduct object detection. In the following section, the three core modules for FR-Net are discussed. Depthwise–pointwise convolution construction is introduced in Section 3.1. The coarse module to guide the search for objects is explained in Section 3.2. Finally, we demonstrate how the object detection module works. 

### 3.1. Depthwise–Pointwise Convolution

**Figure 3:**
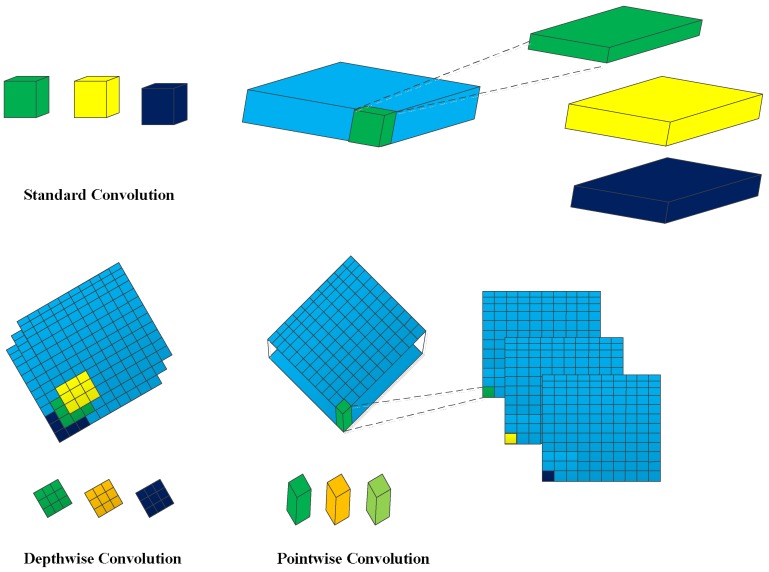
Standard convolutional filters in the top row are replaced by two layers: depthwise convolution in the bottom row, first column, and pointwise convolution in the bottom row, second column.

**Figure 4:**
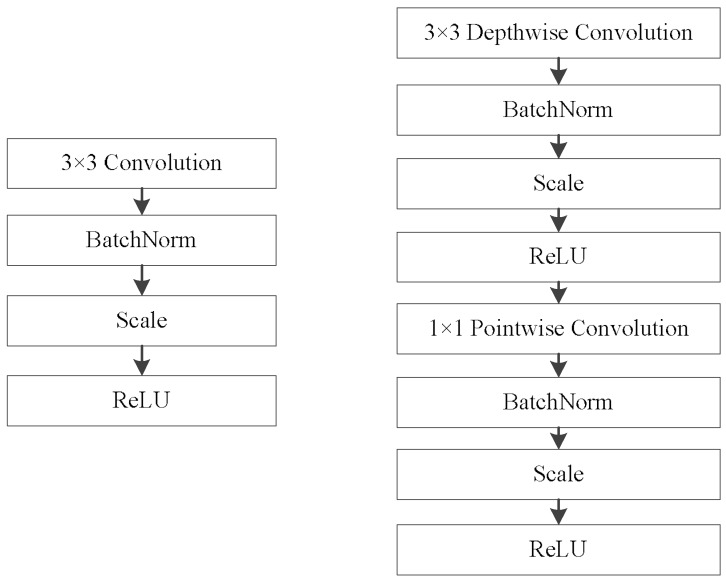
**Left** column denotes the structure of standard convolution, and the **right** column represents the structure of depthwise-pointwise convolution.

It is obvious that a standard convolution operates on both region and channel, which leads to a great amount of calculation. To reduce the computational load, we deploy a depthwise-pointwise convolution, as in Ref. [19]. The depthwise convolution applies a single filter to each input channel, and the pointwise convolution then applies a 1×1 convolution to combine the outputs of the depthwise convolution. The depthwise-pointwise convolution splits a standard convolution into two layers, a depthwise convolution and a pointwise convolution. As shown in Figure 3, the first row demonstrates the operation mechanism of a standard convolution filter, and the bottom row indicates how a standard convolution is factorized into a depthwise convolution and a pointwise convolution. Figure 4 demonstrates a layer with regular convolutions, batch norm, scale, and rectified linear unit (ReLU) nonlinearity to the factorized layer with depthwise convolution and pointwise convolution, as well as batch norm, scale, and ReLU after each convolutional layer.

Assuming that the number of input channels for input feature map is M and the kernel size of the filters is $K_{w}\times K_{h}$ with N channels, the standard convolution operation outputs an $F_{w}\times F_{h}\times N$ feature map, where $F_{w}$ and $F_{h}$ represent the width and the height of the output feature map, and N denotes its channels. A comparison of the computation cost of a standard convolution and a depthwise-pointwise convolution is as follows.

(1) $$L_{st}=K_{w}\times K_{h}\times M\times F_{w}\times F_{h}\times N$$

(2) $$L_{dp}=K_{w}\times K_{h}\times F_{w}\times F_{h}+M\times F_{w}\times F_{h}\times N$$

(3) $$\frac{L_{dp}}{L_{st}}=\frac{K_{w}\times K_{h}\times F_{w}\times F_{h}+M\times F_{w}\times F_{h}\times N}{K_{w}\times K_{h}\times M\times F_{w}\times F_{h}\times N}=\frac{1}{M\times N}+\frac{1}{K_{w}\times K_{h}}$$

In Equations (1)–(3), $L_{st}$ and $L_{dp}$ represent the computational cost of a standard convolution and a depthwise-pointwise convolution, respectively. The result of Equation (3) is the reduction computation. We use 3×3 depthwise convolutions, which can obtain eight to nine times less computation than standard convolution, with only a small reduction in accuracy [43].

The body architecture of the base network is shown in Table 1. All of the layers are followed by a batch norm and ReLU nonlinearity. Figure 4 demonstrates a layer with regular convolutions, batch norm, scale and ReLU nonlinearity to the factorized layer with depthwise convolution, and pointwise convolution, as well as batch norm, scale, and ReLU after each convolutional layer. Down sampling is conducted by stride convolution in the depthwise convolutions, as well as in the first layer, where s1 and s2 represent that the stride steps of the convolutions are 1 and 2, respectively. Counting depthwise and pointwise convolutions as separate layers, the base network has 29 layers. We add convolution feature layers of conv14_1 and conv14_2 to the end of the network to allow for predictions of detections at multiple scales.

### 3.2. Coarse Detection Module

As with the region proposal stage of Faster rcnn, the coarse detection module is introduced to select positive examples preliminarily in dense bounding boxes and initialize the locations coarsely for a better regressor, which estimates the probability of object or not object for each anchor. Particularly, we associate *n* anchor boxes with each cell of the feature map. Each cell is regularly divided on the feature map, and each anchor box has a fixed initial position relative to its corresponding cell. Thus, *n* prior anchor boxes can be obtained at each feature map cell. Each feature map is associated with one specific scale of anchors and the three aspect ratios, i.e., 0.5, 1.0, and 2.0. The first regression is used to predict four offsets of these boxes and the second the confidence probability indicating objects or not objects in these boxes. We obtain two class scores and the four coarse offsets of objects corresponding to the prior anchor boxes. This procedure provides coarse object classifications and locations for subsequent regression, as shown in Figure 1. To tackle the class imbalance issue, we design a rule to filter many well classified negative anchors. Thus, the prior positive anchors and negative anchors with confidence scores that are more than 0.99 are passed to the object detection module. The threshold value of 0.99 is obtained empirically. Different from the region proposal network (RPN) in Faster-rcnn, different feature maps are used to generate anchors with different ratios as well as conventional SSDs. The feature maps whose receptive fields are 8×8, 16×16, 32×32, and 64×64 are selected for the coarse detection module to generate multiscale proposals. In conclusion, this module handles class imbalance at the same time and provides prior information for the object detection module, which further generates object classification and more-accurate location. Furthermore, it reduces the searching space for detecting objects.

### 3.3. Object Detection Module

**Figure 5:**
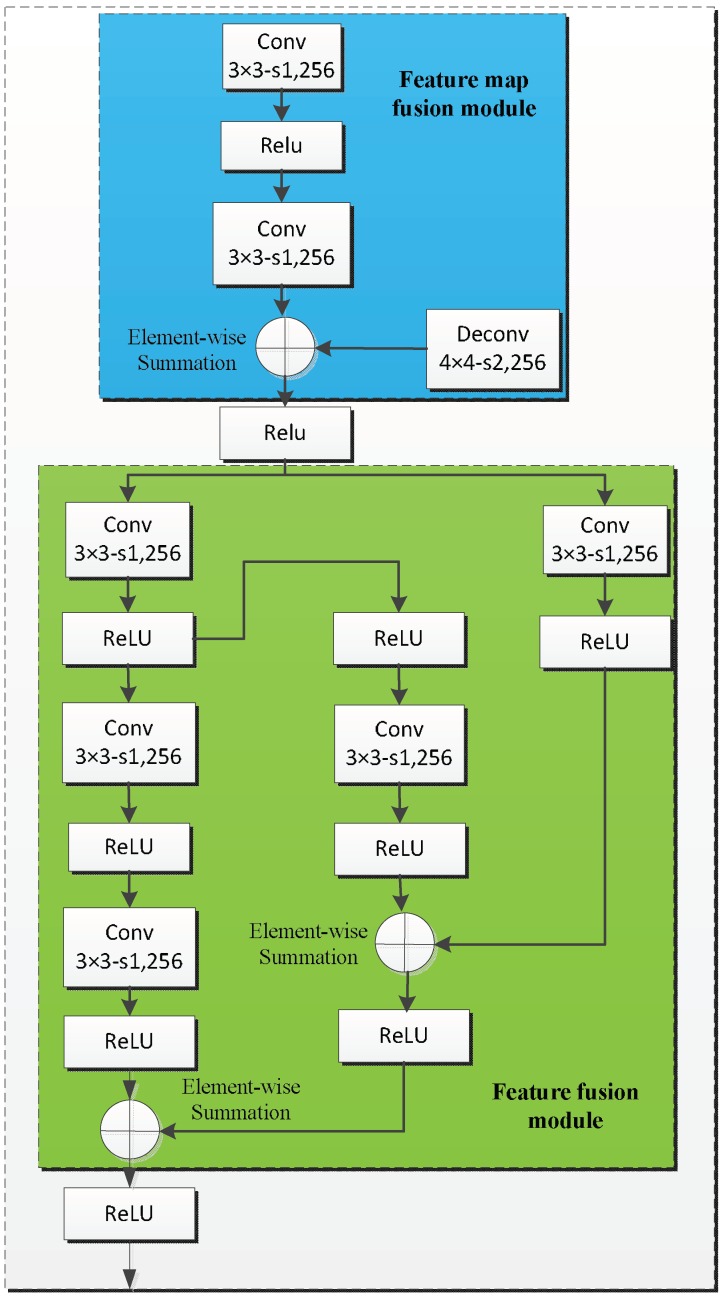
Object detection module.

Some researchers [19,43] have proved that adding the high-level information to integrate large-scale features can improve detection performance, particularly for small objects. As shown in Figure 2, the object detection module shares features with the coarse detection module. However, the object detection module consists of two submodules, i.e., the feature map fusion module and the feature fusion module, which are shown in Figure 5. Inspired by the mechanism of integrating features in DSSD [19], we constructed the feature map fusion module to fuse the feature maps of different layers from the coarse object detection module. As shown in Figure 5, this module makes interaction between adjacent feature maps and enriches the semantic information of former layers, where conv 3 × 3-s1, 256 denotes that the size of the filter is 3 × 3, the number of the filter is 256, and the step of the convolution is 1. We use the deconvolution operation in order to ensure the different feature maps at the same dimension, and we adopt elementwise summation to merge the corresponding two feature maps together. Then, the fusion feature module incorporates context by enlarging the window around the candidate proposals. We add the context information by means of simple convolution layers. 

As with the approach in Ref. [44], we adopted sequential 3×3 filters instead of larger convolutional filters to reduce the number of parameters, i.e., three 3×3 filters constitute a 7×7 filter. By this way, we can increase the receptive field of the corresponding layer, as well as the object scale during the detection stage, which results in more efficiency in detecting small objects. As well as the feature map fusion module, elementwise operation was utilized to fuse different features together. Our object detection module has better feature fusion performance than DSSD. For the proposed method, we have four object detection modules that are corresponding to feature maps of different scales. Finally, the object detection calculates *c* (i.e., six classes of railway traffic objects are detected) class scores and four accurate offsets of objects relative to the prior anchor boxes proposed by the coarse detection module, yielding *c* + 4 outputs for each prior anchor to complete the detection. 

## 4. Training

We used stochastic gradient descent with momentum and weight decay for training the network. To expand the existing datasets and to construct a more robust model, we used several strategies for data augmentation [21]. As with SSD, we randomly selected one patch with the following options: the original image, patches of expanding or cropping the original image with different Jaccard overlap, and patches of random photometric distortion and flipping. Moreover, we used the center code type to encode the bounding boxes and the same matching strategy and hard negative mining strategy, as well as SSD. More details can be found in Ref. [21]. 

FR-Net has a multitask loss. This loss can be formulated, as follows.

(4) $$\begin{array}{ll} L(\{p_{i}\},\{c_{i}\},\{t_{i}\},\{d_{i}\}) & =\frac{1}{N_{c}}\left(\sum_{i}L_{c}(p_{i},l_{i})+\sum_{i}I(l_{i}\geq 1)L_{r}(c_{i},g_{i})\right)\\ & +\frac{1}{N_{d}}\left(\sum_{i}L_{d}(t_{i},l_{i})+\sum_{i}I(l_{i}\geq 1)L_{r}(d_{i},g_{i})\right) \end{array}$$

where *i* is the index of the anchor in a minibatch, $l_{i}$ denotes the ground truth class label of anchor *i*, and $g_{i}$ is the ground truth location of anchor *i*. $p_{i}$ and $c_{i}$ are the predicted probability of the anchor *i* being an object and the coarse coordinates offset of the prior anchor *i* in the coarse detection module, respectively. $t_{i}$ and $d_{i}$ are the predicted object class and coordinates of the prior anchor box in the object detection module, respectively. $N_{c}$ and $N_{d}$ are the number of positive anchors in the coarse detection module and in the object detection module, respectively. The classification loss $L_{c}$ is the cross-entropy loss over two classes (object or not) and the classification loss $L_{d}$ is the softmax loss over the confidence scores of multiple classes. $L_{r}$ represents the bounding box regression loss. As in Ref. [20], we parameterize the regression space with a log-space shift in the box dimensions and a scale invariant translation and use smooth $l_{1}$ loss as $L_{r}$. I(·) is the indicator function that limits the regression loss only to the positively assigned anchors, i.e., $l_{i}\geq 1$ represents that the anchor is positive, and 0 otherwise.

## 5. Experiment and Results

We comprehensively evaluated our method on the railway traffic datasets. In Section 5.1, we show the process of making railway traffic datasets by collecting real-word railway traffic videos. To investigate the behavior of the proposed method, we conducted several experiments that were based on the railway traffic datasets. To test the performance of our method, we compared it with the classic one-stage approach SSD, DSSD, and two-stage approach Faster rcnn. All of the experiments were conducted on a Caffe platform with a backbone of VGG16, except for DSSD. The backbone of DSSD was ResNet-101, followed by its respective paper [38]. We analyze the effectiveness of different components of FR-Net in an ablation study.

### 5.1. Datasets

To ensure the diversity of sampling images, we collected data in different weather conditions, light conditions, and run states of a train by the railway object detection system (see Figure 1). To ensure the diversity of the data, we obtained pictures in different weather conditions, different lighting conditions, and different speed conditions. Then, we converted a series of railway traffic videos into a sequential frame of images. Because of the similar content between adjacent sequences, we sampled the image every five frames. In total, 7342 sample images with the size of 640 pixel × 512 pixel were collected. When considering the railway shape and the possible obstacles in the process of train scheduling, we labeled the images with six classes: bullet train, pedestrian, railway straight, railway left, railway right, and helmet. We took 83% of these images for training and validation, and the rest for testing. In particular, the railway tracks (Railway Straight, Railway Left, and Railway Right) at the center of the field of view were labeled according the observation habituation of human vision. We take 70% of these images for training and validation, and the rest for test. The number of each class in the dataset is as shown in Table 2.

### 5.2. Effectiveness Performance

The backbone network in our experiments was VGG16 [20] and it was pretrained on the ILSVRC-LOC dataset [43]. The input sizes of the testing images were 320×320. FC6 and FC7 of VGG16 were substituted by depthwise–pointwise convolution layers using subsampling parameters as DeepLab-LargeFOV [45] did. Here, feature maps with sizes of 40×40, 20×20, 10×10, and 5×5 were used to detect objects in multiple scales. Some hyper parameters were set as follows. We set the default batch size to 32, optimization method to SGD with 0.9 momentum and 0.0005 weight decay, and initial learning rate to 0.001. The maximum number of iterations of all the experiments was 200,000.

#### Comparison with State-of-Art

We used average precision (AP) to evaluate the model effectiveness. Usually, AP is calculated by the area of a curve that was composed of recall rate and precision rate. We compared FR-Net with the state-of-the-art detectors SSD, DSSD, and Fast-rcnn. 

The comparison results with the state-of-art detectors are as shown in Table 3. With input size of 320×320, FR-Net produces 0.8938 mAP without bells and whistles, which is much better than several modern optimization methods. For Faster rcnn, the experimental results are not very good. In our opinion, the reason for this may be that Faster rcnn may be more suitable for large images, because the input size of Faster rcnn is 1000 × 600. However, the size of the railway images that were collected was 640 × 512, and the reverse interpolation may result in losing image information. The performance of DSSD was also acceptable. However, the model size of DSSD (see the sixth column of Table 1) was too large to transplant into a mobile device.

**Figure 6:**
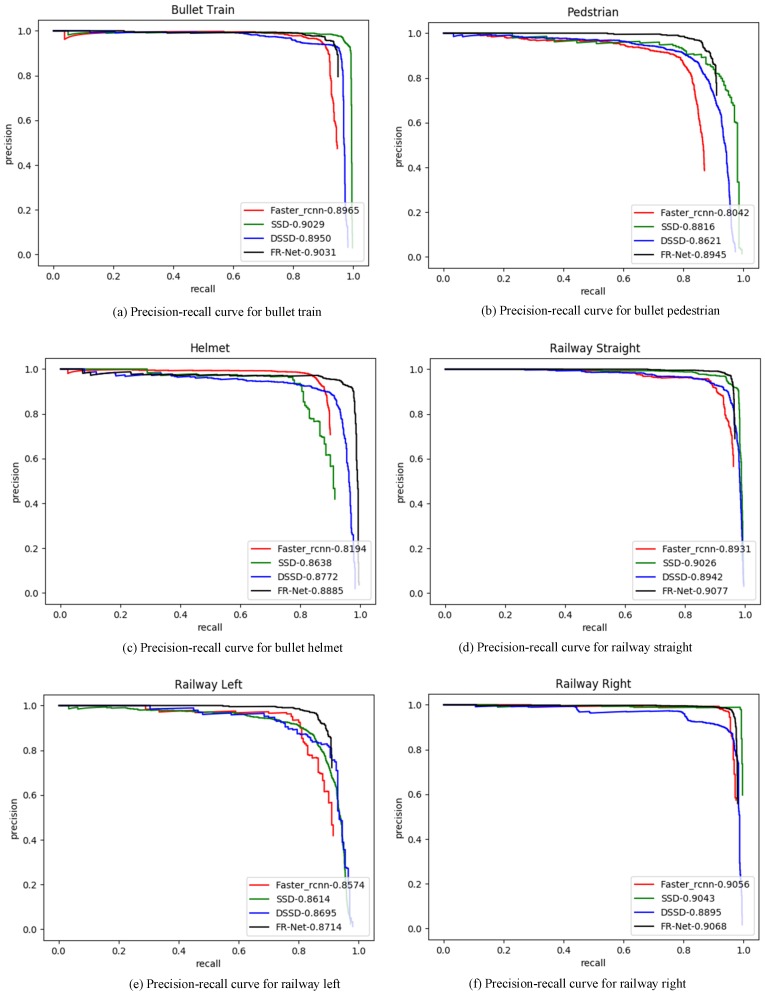
Precision-recall curves for different optimization algorithms with respect to different objects. The red line represents the result of Faster rcnn. The green line reflects the result of the single-shot detector (SSD) method. The blue line shows the result of the deconvolutional single-shot detector (DSSD). FR-Net is the black line.

The precision–recall curves on the railway traffic datasets are presented in Figure 6. We demonstrate the recall–precision curves of the three typical railway traffic obstacles, i.e., bullet train, pedestrian, and helmet, as shown in Figure 6a–c. FR-Net is superior to the other three methods and it obtains the highest AP value of the three classes. Especially, for the recall–precision curve of the helmet in Figure 6c, FR-Net achieves the highest AP, which is 2.5% larger than SSD. However, Faster rcnn obtains the lowest AP for detecting the small target of a helmet. For the recall-precision curve of railway left in Figure 6e, FR-Net obtains the lowest AP of 0.8714 when compared to the other five classes. It can be seen from Table 2 that railway left has the minimum number of samples. However, the number of railway left and right is less than railway straight in the actual scene. The detection of railway right and left are used to remind the driver of the train to pay attention to the safe driving ahead. Figure 6 shows that FR-Net achieves state-of-the-art results on the railway traffic dataset. Moreover, the results imply that FR-Net can detect large and small railway obstacles effectively.

### 5.3. Runtime Performance

We present the runtime performance of FR-Net and the state-of-the-art methods in the fifth column of Table 3. The real-time performance is evaluated on a machine with GeForce GTX1080Ti (NVIDIA Corporation, Santa Clara, CA, USA), CUDA 8.0, and cuDNN v6. The FR-Net processes an image in 13.66 ms (72.3 FPS), with input sizes of 320 × 320 pixels, while SSD consumes 21.28 ms (47 FPS) with input sizes of 300 × 300 pixels. It is 7.23 times faster than Fast rcnn with two-stage detection, and 1.54 times faster than SSD with one-stage detection. Moreover, it is 10 times faster than DSSD using the feature fusion method. DSSD yields the worst real-time performance and has the largest model size among the mentioned methods. The model size of FR-Net is 24.7% less than SSD, which meets the requirements of most mobile devices. 

**Figure 7:**
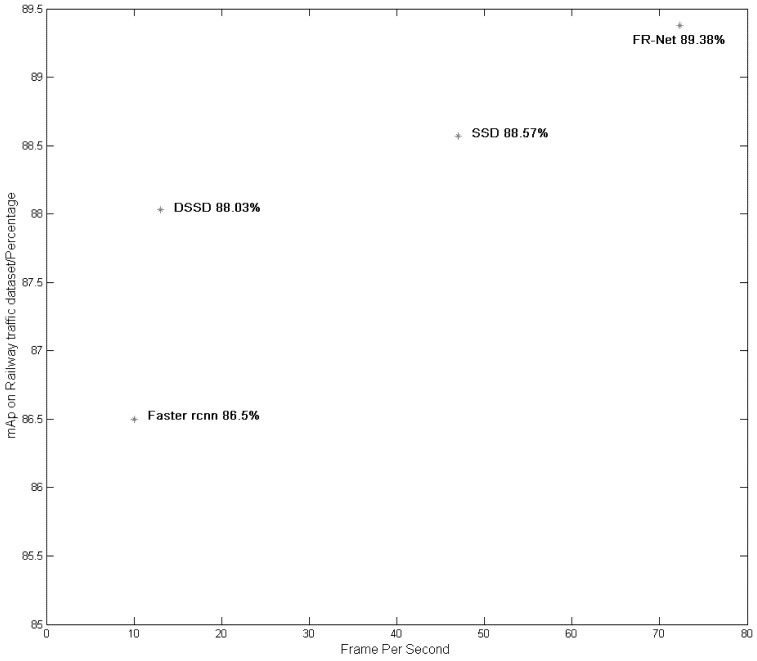
Speed and accuracy distribution with different methods. All the speeds were measured on Nvidia GeForce GTX1080Ti.

We present the comprehensive evaluation results in Figure 7. FR-Net achieves the fastest speed when compared with the other three methods, with the highest mAP. Our FR-Net results in a good tradeoff, both in effectiveness and real time performance.

### 5.4. Visual Results of Small-Object Detection

**Figure 8:**
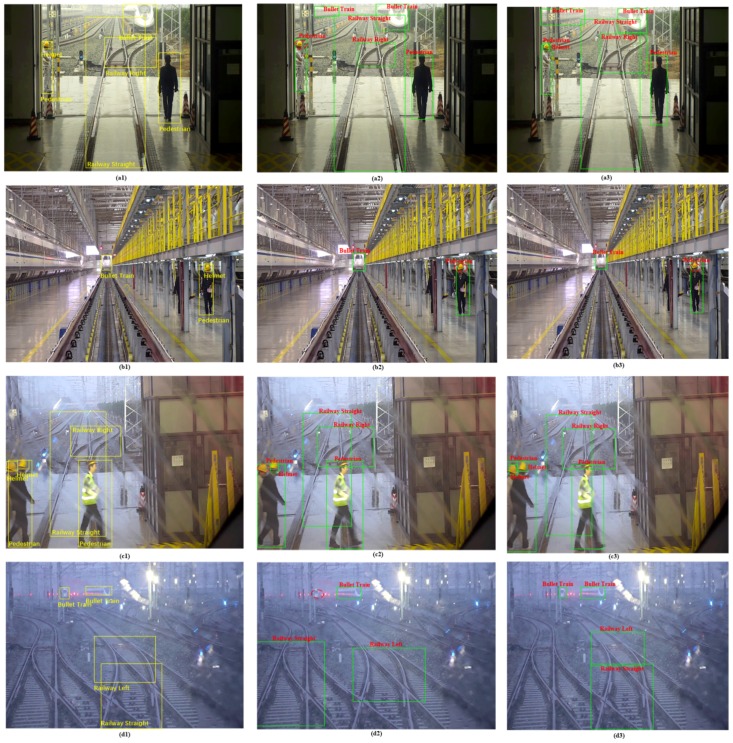
FR-Net vs. SSD300. All three models were trained with the railway dataset that was mentioned above. The left column represents the original detection images and the yellow rectangles denote the ground truth of the objects. The middle column contains the results from the conventional SSD300, and the right column is from the proposed method. The **first row** denotes scenario of object detection of outside train garage, the **second row** denotes object detection in repair scence of train garage, the **third row** represents pedestrain detection of train garage, the **fourth row** denotes object detection during driving train. Bounding boxes with a score of 0.8 or higher are drawn. The red dotted line represents the category that was not detected.

In this section, we focus on testing FR-Net for detecting small objects. To evaluate the performance of the network for small-object detection, we set the category threshold score to 0.8, which means that the bounding boxes with a score of 0.8 or higher are drawn. For a better view on screen, we re-edited the category manually and omitted the scores of each category. Here, we compare the results with the classic SSD-300 method. As shown in Figure 8, the left column (see Figure 8a1,b1,c1) represents the original detection images and the yellow rectangles denote the ground truth of the objects, and the bottom column is from our method. For SSD300, Figure 8a2,b2,c2, we can see that the helmets have a different extent of detection failure. In Figure 8d2, the bullet train is not detected and the leftmost railway of the field of view is wrongly detected. For the proposed FR-Net, the object bounding boxes were obtained by two-step regression, i.e., the coarse detection module coarsely refined the locations and the sizes of prior anchors to provide better initialization for the subsequent module and reduced search space for the classifying, so that the object detection module could detect a small target with the prior anchors. However, the result of Figure 8b3 demonstrates that we still have much room for improving the performance of FR-Net for small objects. 

### 5.5. Robustness Test

**Figure 9:**
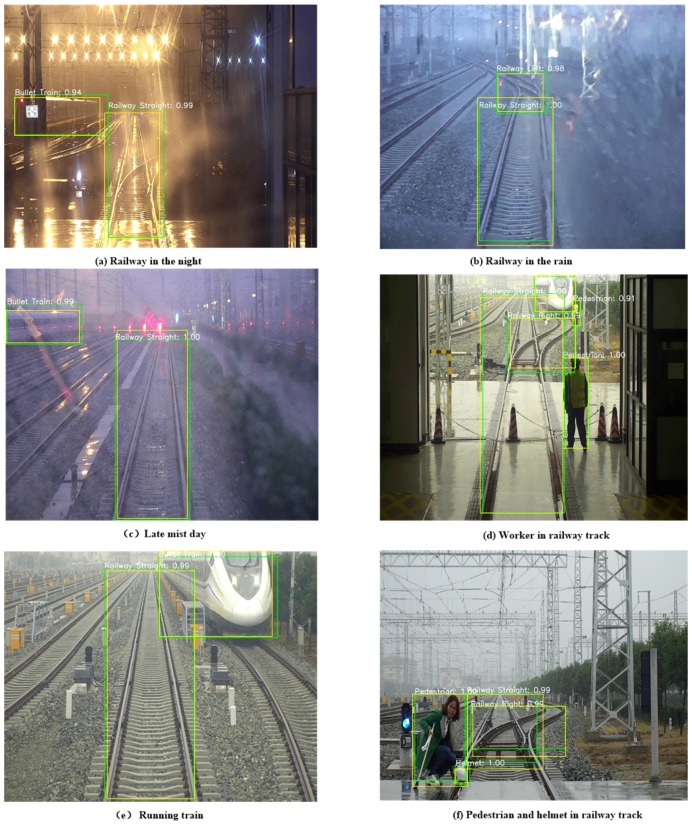
Robustness test in different environmental conditions.

The robustness of the proposed method to different environmental conditions is addressed in this section. The yellow boxes represent the ground truth boxes of the objects, as well as Figure 8. The experiment results are shown in Figure 9a–f. Figure 9a–c shows that poor-quality images were acquired due to the due to bad weather. However, the proposed method detects the curves or straight railway with high classification scores, as in i.e., Figure 9b. Our proposed FR-Net can detect obstacles ahead well in order to confirm whether the front railway line is occupied in the night and mist days, and to ensure that the train is running safely, as shown in Figure 9a,c. Figure 9d,f illustrate that FR-Net can detect pedestrians crossing the railway and obstacles in the railway with good performance. In particular, when detecting obstacles straight ahead, our equipment (see in Figure 1) sends a voice alarm to remind the driver of the train to ensure safety. The result of running the train in Figure 9e shows that FR-Net focuses on detecting the railway track on which the train runs. Moreover, the result shows that the proposed method can reduce unnecessary detection and can be suitable for human vision. Although some images are in low quality, the FR-Net still achieves considerable detection results. The robustness experiment results show that FR-Net can meet the needs of practical applications in shunting mode.

### 5.6. Ablation Study

To demonstrate the performance of different components for FR-Net, we constructed three experiments. We used the same parameter setting and input size (320×320) in the evaluation, except in the third experiment. The third experiment evaluated the performance with respect to different input sizes. All of the models were trained and tested on the railway traffic dataset.

#### 5.6.1. Comparison of Various Designs

To illustrate the use of the coarse object detection module and the object detection module with feature fusion effectively, we introduced SFR-Net, which is FR-Net without the depthwise and pointwise convolution modules and is shown in the third column in Table 4. Mobile-Net was designed without the coarse object detection module and the object detection module, as well as FR-Net. The performances of various designs are shown in Table 5. The result shows that SFR-Net achieved the best results among the six classes. The AP value for small objects, such as helmets, increased by 7.5% and 1% when compared with Mobile-Net and FR-Net, respectively. Mobile-Net obtained the fastest speed with the lowest mAP. However, FR-Net achieved excellent performance both in AP and real time, with little drop in mAP. FR-Net provided a good compromise both in the effectiveness and real-time performance.

#### 5.6.2. Analysis of mAP and Recall vs. IOU

**Figure 10:**
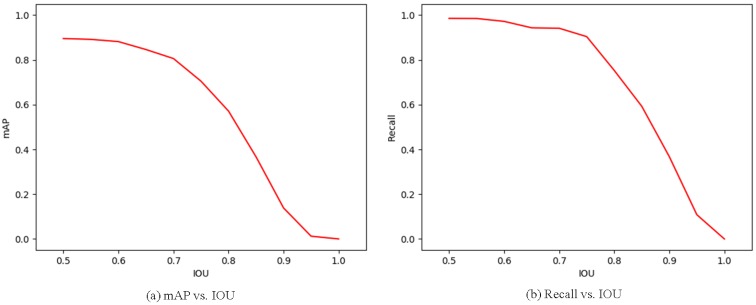
mAP and recall vs. intersection over union (IOU) overlap ratio on the railway traffic test set.

We computed the mAP and the recall of FR-Net at different intersection over union (IOU) ratios with ground truth boxes. The mAP and recall for the IOU metric are loosely related to the ultimate detection accuracy. IOU reflects the object location accuracy in images to some extent. By using this metric, we found the balance point between object location accuracy and detection accuracy. As shown in Figure 10, both the mAP and recall descended with the increase of IOU. When the IOU reached 0.55, the mAP was 0.9849, and the recall was 0.8916, which are acceptable in use. To achieve a tradeoff in location accuracy and detection accuracy, we set IOU below 0.55 in practical application.

#### 5.6.3. Performance with Respect to Different Input Sizes

As shown in Table 6, the input size significantly influenced the detection performance. FR-Net-512 detected the railway traffic objects more effectively than FR-Net-320. The reason is that high-resolution inputs can enlarge the small objects for the model to detect them effectively. However, increasing the input size can improve the performance for detecting small objects; it will be a burden on real-time inference. FR-Net-320 achieved a good tradeoff in both effectiveness and real-time performance.

## 6. Conclusions

In this research, we proposed an automatic object detection system that is based on FR-Net to tackle the real-word railway traffic object detection issue in shunting mode. To account for effectiveness and efficiency, three novel parts were introduced in FR-Net, including depthwise convolution, the coarse detection module, and the object detection module. We replaced all of the standard convolutions with depthwise-pointwise convolutions, except for the first layer for efficiency. The coarse object detection module provided prior anchors for the object detection module, which is a similar process to the default boxes used in SSD. Taking the prior anchor boxes as input, the object detection module obtained sufficient feature information using two submodules (i.e., the feature map fusion module and feature fusion module) for object detection, which led to more-accurate detection results, especially for small targets. Depthwise convolution was used for efficiency, whereas the other two modules were responsible for effectiveness. Several experiments on railway traffic datasets were conducted, and the results show that FR-Net achieves 0.8953 mAP with 72.3 FPS performance on a machine with a GeForce GTX1080Ti. The experiments on robustness for different environment conditions and small-object detection showed that FR-Net exhibited good performance for railway obstacle detection. However, there is still much room to improve the performance of FR-Net for small-obstacle detection. The evaluation of different components for FR-Net demonstrates that the proposed method achieves a good tradeoff in both effectiveness and real-time performance.

In the future, we plan to expand the application of FR-Net to detect objects in some other specific situations or to transplant it into some special platform, i.e., an embedded system. Furthermore, we will conduct further research to improve detection performance.

## Figures and Tables

**Table 1:** The body architecture of the base network.

Type/Stride	Filter Shape	Input Size
Conv0/s2	3 × 3 × 3 × 32	320 × 320 × 3
Conv1 dw/s1	3 × 3 × 32 dw	160 × 160 × 32
Conv1/s1	1 × 1 × 32 × 64	160 × 160 × 32
Conv2 dw/s2	3 × 3 × 64 dw	160 × 160 × 64
Conv2/s1	1 × 1 × 64 × 128	80 × 80 × 64
Conv3 dw/s1	3 × 3 × 128 dw	80 × 80 × 128
Conv3/s1	1 × 1 × 128 × 128	80 × 80 × 128
Conv4 dw/s2	3 × 3 × 128 dw	80 × 80 × 128
Conv4/s1	1 × 1 × 128 × 256	40 × 40 × 128
Conv5 dw/s2	3 × 3 × 128 dw	40 × 40 × 256
Conv5/s1	1 × 1 × 256 × 256	40 × 40 × 256
Conv6 dw/s2	3 × 3 × 256 dw	40 × 40 × 256
Conv6/s1	1 × 1 × 256 × 512	20 × 20 × 256
Conv7 dw/s1	3 × 3 × 512 dw	20 × 20 × 512
Conv7/s1	1 × 1 × 512 × 512	20 × 20 × 512
Conv8 dw/s1	3 × 3 × 512 dw	20 × 20 × 512
Conv8/s1	1 × 1 × 512 × 512	20 × 20 × 512
Conv9 dw/s1	3 × 3 × 512 dw	20 × 20 × 512
Conv9/s1	1 × 1 × 512 × 512	20 × 20 × 512
Conv10 dw/s1	3 × 3 × 512 dw	20 × 20 × 512
Conv10/s1	1 × 1 × 512 × 512	20 × 20 × 512
Conv11 dw/s1	3 × 3 × 512 dw	20 × 20 × 512
Conv11/s1	1 × 1 × 512 × 512	20 × 20 × 512
Conv12 dw/s2	3 × 3 × 512 dw	20 × 20 × 512
Conv12/s1	1 × 1 × 512 × 1024	10 × 10 × 512
Conv13 dw/s1	3 × 3 × 1024 dw	10 × 10 × 1024
Conv13/s1	1 × 1 × 1024 × 1024	10 × 10 × 1024
Conv14_1/s1	3 × 3 × 1024 × 256	10 × 10 × 1024
Conv14_2/s2	3 × 3 × 256 × 512	10 × 10 × 256

**Table 2:** The number of each class in the dataset.

Class	Number
Bullet Train	3671
Pedestrian	9371
Railway Straight	3863
Railway Left	652
Railway Right	1804
Helmet	3089

**Table 3:** Comparison results with the state-of-art on the railway traffic datasets.

Method	Backbone	Input Size	Boxes	FPS	Model Size (M)	mAP (%)
SSD	VGG-16	~300×300	8732	47	98.6	0.8861
Faster-RCNN	VGG-16	~1000×600	300	10	521	0.8632
DSSD-321	ResNet-101	~321×321	17,080	13	623.4	0.8813
FR-Net-320	VGG-16	~320×320	6375	72.3	74.2	0.8953

**Table 4:** Models of various designs.

Component	Mobile-Net	SFR-Net	FR-Net
Depthwise–pointwise?	√	-	√
The coarse object detection modules?	-	√	√
The object detection module?	-	√	√

**Table 5:** Performance of various designs. All of the models are trained on the railway traffic dataset.

Method	mAP(%)	FPS	Bullet Train	Pedestrian	Railway Straight	Railway Left	Railway Right	Helmet
Mobile_Net	0.8692	106	0.8891	0.8315	0.9012	0.8628	0.9069	0.8239
SFR-Net	0.8997	26.1	0.9067	0.8933	0.9071	0.8841	0.9075	0.8994
FR-Net	0.8953	72.3	0.9031	0.8945	0.9077	0.8714	0.9068	0.8885

**Table 6:** Performance with respect to different input sizes.

Method	mAP(%)	FPS	Bullet Train	Pedestrian	Railway Straight	Railway Left	Railway Right	Helmet
FR-Net-512	0.9046	43.2	0.9046	0.9017	0.9060	0.9007	0.9073	0.9075
FR-Net-320	0.8953	72.3	0.9031	0.8945	0.9077	0.8714	0.9068	0.8885
