# Correction: Ye, T.; et al. Automatic Railway Traffic Object Detection System Using Feature Fusion Refine Neural Network under Shunting Mode. *Sensors* 2018, *18*, 1916

**DOI:** 10.3390/s19143044

**Published:** 2019-07-10

**Authors:** Tao Ye, Baocheng Wang, Ping Song, Juan Li

**Affiliations:** 1 Beijing Institute of Remote Sensing and Equipment, 52 Yongding Road, Haidian District, Beijing 100039, China; 2 College of Computer and Science Technology, North China University of Technology, 5 Jin Yuan Zhuang Road, Shijingshan District, Beijing 100144, China; 3 School of Instrumentation Science and Opto-Electronics Engineering, Key Laboratory of Precision Opto-Mechatronics Technology, Ministry of Education, Beihang University, Beijing 100191, China

The authors wish to make the following corrections to this paper [1].

We have found three inadvertent errors in our paper published in this journal [1]:

1. In Section 5.1 of this paper [1], the sentence, “We take 70% of these images for training and validation, and the rest for test” should be removed, as it conflicts with line 305, the sentence, “We take 83% of these images for training and validation, and the rest for test”. We intend to delete the sentence, “We take 70% of these images for training and validation, and the rest for test”. This modification is necessary in the revised manuscript to correct the error. We therefore added the sentence, “We take 83% of these images for training and validation, and the rest for test” in the revised manuscript, following the reviewers’ advice, and did not delete the sentence, “We take 70% of these images for training and validation, and the rest for test” in the revised manuscript.

2. In Section 5.2 of this paper [1], the sentence “We demonstrate the recall–precision curves of the three typical railway traffic obstacles, i.e., bullet train, pedestrian, and helmet, as shown in Figure 6a–c. FR-Net is superior to the other three methods and obtains the highest AP value of the three classes.” This sentence should be modified to state: “We demonstrate the recall–precision curves of the three typical railway traffic obstacles, i.e., bullet train, pedestrian, helmet, railway straight, railway left, and railway right, as shown in Figure 6a–f. FR-Net is superior to the other three methods and obtains the highest AP value of the six classes.”

In the original paper, we make AP comparisons for three classes. However, we added the other three classes, i.e., railway straight, railway left, and railway right in the revised manuscript, following the reviewers’ suggestions. We may not have included some expression modification in the revised manuscript, such as Figure 6a–c, which will change to Figure 6a–f.

These changes have no material impact on the conclusion of our paper. We apologize to our readers.
